# Patterns of childhood adverse events are associated with clinical characteristics of bipolar disorder

**DOI:** 10.1186/1471-244X-13-97

**Published:** 2013-03-22

**Authors:** Sara Larsson, Monica Aas, Ole Klungsøyr, Ingrid Agartz, Erlend Mork, Nils Eiel Steen, Elizabeth A Barrett, Trine V Lagerberg, Jan Ivar Røssberg, Ingrid Melle, Ole A Andreassen, Steinar Lorentzen

**Affiliations:** 1 Institute of Clinical Medicine, University of Oslo, Oslo, Norway; 2 Division of Mental Health and Addiction, Oslo University Hospital, Oslo, Norway; 3 Department of Psychiatric Research, Diakonhjemmet Hospital, Oslo, Norway; 4 National Centre for Suicide Research and Prevention, Institute of Clinical Medicine, University of Oslo, Oslo, Norway; 5 Institute of Clinical Medicine, Clinic of Health and Addiction, University of Oslo, 1039, Blindern, Oslo, N-0315, Norway

**Keywords:** Childhood trauma, Bipolar disorder, Clinical characteristics

## Abstract

**Background:**

Previous studies in bipolar disorder investigating childhood trauma and clinical presentations of the illness have mainly focused on physical and sexual abuse. Our aim was to explore further the relationship between childhood trauma and disease characteristics in bipolar disorder to determine which clinical characteristics were most strongly associated with childhood trauma total score, as well as subtypes of adverse childhood events, including physical, sexual, emotional abuse and neglect.

**Methods:**

141 Patients with bipolar disorder were consecutively recruited, and disease history and clinical characteristics were assessed. History of childhood abuse was obtained using the Childhood Trauma Questionnaire (CTQ). Statistical methods used were factor analysis, Poisson and linear regression, and generalized additive modeling (GAM).

**Results:**

The factor analysis of CTQ identified three factors: emotional abuse/neglect, sexual abuse and physical abuse. There were significant associations between CTQ total score and earlier onset of illness, reduced level of psychosocial functioning (GAF; Global Assessment of Functioning) and decreased number of hospitalization, which mainly were due to the factor emotional abuse/neglect. Physical abuse was significantly associated with lower GAF scores, and increased number of mood episodes, as well as self-harm. Sexual abuse was significantly associated with increased number of mood episodes. For mood episodes and self-harm the associations were characterized by great variance and fluctuations.

**Conclusions:**

Our results suggest that childhood trauma is associated with a more severe course of bipolar illness. Further, childhood abuse (physical and sexual), as well as emotional abuse and neglect were significantly associated with accelerating staging process of bipolar disorder. By using specific trauma factors (physical abuse, sexual abuse and emotional abuse/neglect) the associations become both more precise, and diverse.

## Background

Traumatic events in childhood have been proposed as important contributors to psychiatric disorders. Although a history of childhood trauma is observed across populations, childhood trauma events are more frequently reported in patients with psychiatric disorders [1-4].

In bipolar disorder, there are indications that childhood trauma is associated with clinical characteristics [5], including earlier onset of the illness [6], a rapid cycling course [6], more psychotic features [7-9], higher number of lifetime mood episodes [10-12], as well as suicide ideation and attempts [13]. However, there are discrepant findings, and the patterns of clinical characteristics across different symptom domains have not yet been thoroughly investigated.

The knowledge about how specific types of trauma are associated with clinical characteristics is still sparse. Childhood physical abuse seems to be the strongest predictor of unfavorable clinical characteristics in bipolar disorder; however, very few studies have paid interest to emotional abuse and neglect [14]. Interestingly, there is evidence that subtypes of trauma, particularly emotional abuse [5,15] is increased in patients with bipolar disorder. Indeed, higher prevalence of emotional abuse in patients with bipolar disorder compared to healthy controls has been reported, even after controlling for other types of abuse [5,15].

There are several methodological challenges in this line of research: differences in the methods used to assess childhood trauma (structured interview versus self–report measures) may cause problems in comparing different studies [5,16]. Furthermore, some studies present total trauma scores, while others have focused on specific types of abuse, mainly physical or sexual abuse [5,17]. Another challenge may be that study samples may consist of patients that have been solicited or who have consulted highly specialized clinics, which will impact the representability of the results.

The overall aim of this study was to obtain a more consistent picture of the association between childhood adverse events and disease characteristics in bipolar disorder, including measures of emotional abuse and neglect, which has been sparsely investigated in prior studies. Specific aims: 1. Determine if childhood trauma is associated with more serious disease traits, indicating worse outcome, by earlier age at onset, number of illness episodes and hospitalizations. 2. Determine if childhood trauma is associated with worse current functioning and symptom level measured by GAF (Global Assessment of Functioning). 3. Determine if *specific trauma types* are differentially associated with the clinical characteristics.

## Method

### Participants

This study is part of an ongoing study of severe psychiatric disorders (Thematically Organized Psychosis (TOP) Study), where patients are recruited consecutively from psychiatric in- and out-patient units at three major hospitals in Oslo providing health care for the entire city of Oslo. Due to the publicly funded, catchment-area organization of the health care system in Norway, the sample represents patients from all socio-demographic strata. Inclusion criteria were age 18-65 years, a diagnosis of bipolar disorder according to DSM-IV, and ability to give informed consent. Exclusion criteria were history of severe head trauma, mental retardation, neurological disorder, and lack of knowledge of any Scandinavian language.

It is a current ongoing debate whether borderline personality disorder (BPD) belongs to the bipolar spectrum [18]. We know from the literature that borderline patients, similar to bipolar patients, report high levels of childhood trauma [18], as well as emotional dysregulation. In our study, however, we only included patients who met a DSM-IV SCID 1 diagnosis of Bipolar disorder, independent of a possible personality disorders they may also have had. The project was approved by the Regional Committee for Medical Research Ethics and the Norwegian Data Inspectorate. Written informed consent for participating in the study was obtained from all participants.

The Childhood Trauma Questionnaire (CTQ) was completed by 158 patients. Two of these were excluded due to brain injury, 14 patients were excluded due to a neurological disorder, and one was excluded due to an IQ below 70, which left us with a sample of 141 patients for the following analyses. See Table 1 for more details. Of the resulting 141 patients, 103 patients were diagnosed with bipolar I disorder, 26 with bipolar II disorder, and 12 patients with bipolar disorder NOS.

**Table 1:** Demographics and clinical symptom characteristics

**Variable**	**Mean (SD)**
Age	32.4 (11.5)
Age at Illness onset, years	21.8 (9.0)
GAF^1^	
GAF-S	54.3 (12.1)
GAF-F	50.7 (12.5)
PANSS^2^ score	
positive symptoms	10.5 (4.0)
negative symptoms	10.2 (3.6)
CTQ total score	17.5 (15.2)
Years of education	14.0 (13.2)
Gender, n (%)	
Male	56 (40%)
Female	85 (60%)
Depressive episodes	5.4 (11.5)
Manic episodes	2.4 (7.8)
Hypomanic episodes	5.5 (23.4)
Psychotic episodes	1.4 (3.0)
Hospitalizations	1.97 (2.53)

^1^GAF =Global Assessment of Functioning, Symptom (GAF-S) and Function (GAF-F); ^2^PANSS, Positive and Negative Symptom Scale.

### Clinical assessment

Clinical assessment was carried out by trained psychiatrists, MDs or psychiatrists in training, and clinical psychologists. Diagnosis was based on the Structured Clinical Interview for DSM-IV Axis I disorders (SCID-I). Diagnostic reliability was found satisfactory [19] with overall agreement for DSM-IV diagnostic categories of 82% and the overall κ 0.77 (95% CI: 0.60-0.94). Current positive and negative symptoms were rated using the Positive and Negative Symptom Scale (PANSS) [20]. Inter-rater reliability was acceptable with intra-class correlation coefficients [21] for PANSS subscales ranging from 0.71 to 0.73. Participants were defined as currently psychotic if they scored 4 or higher on any one of the following PANSS items: P1, P3, P5, P6, and G9. Age of onset was defined as the age when the subject first met DSM-IV criteria for a major depressive, manic, hypo-manic, or mixed episode. The number of illness episodes was assessed from the SCID-interview and a semi structured clinical interview. Functional level was assessed with the Global Assessment of Functioning Scale, split version-function score; Global Assessment of Functioning (GAF-F), and Symptoms (GAF-S) [22], interrater reliability was good with ICC (1.1) of .86 [23].

The demographic and clinical characteristics of the sample are presented in Table 1.

### Childhood trauma questionnaire (CTQ)

To measure childhood adverse events, we used the Childhood Trauma Questionnaire (CTQ), a retrospective questionnaire yielding scores for the five subscales: childhood physical abuse, emotional abuse, sexual abuse, physical neglect, and emotional neglect [24]. Reliability and validity of the CTQ have been demonstrated previously [24]. In this study the short version (28 item version) of the CTQ translated to Norwegian was used (see [25], enquiring about traumatic experiences in childhood with answers ranging from “never true”, “rarely true”, “sometimes true”, “often true”, to “very often true”. Childhood trauma total score was created, summarizing scores on all sub-scales, which ranged from 0-95.

### Statistical analyses

All statistical analyses were performed with the packages PASW Statistics 18 (Release 18.0.1) and R (Version 2.12.0). Continuous variables are presented as mean ± standard deviation. In order to potentially reduce number of sub-scales and preserve the explained variance, factor analysis was used. The factor structure of the CTQ was analyzed with Principal Axis Factoring method in PASW, with varimax rotation to obtain uncorrelated factors and with Bartlet method of estimating factor scores. The magnitude of the Eigen values for the different factors and a Scree plot was used to suggest an appropriate number of factors, and the simplest structure was chosen.

To assess the association between degree of childhood trauma and age at onset, and different symptoms subsequent to the onset, different regression models were used. A Cox proportional hazards model was used for incidence rate (hazard rate) of the onset of diagnosis, across patients. Poisson regression (log link) was used to model the incidence rates within the patients, of various types of episodes, and finally linear regression was used to model some of the continuous outcomes, like level of functioning.

To assess the potential non-linearities in the associations between continuous covariates (e.g. CTQ total score or factor scores) and the response in the regression models, a generalized additive modeling (GAM) approach was chosen (R Development Core Team 2010). This function performs smoothing spline fits with automatic smoothness selection for an arbitrary number of covariates. An illustrative plot is generated for each smoothed function and how it affects the incidence rate (on the log – scale), with point wise ± 2 × standard error to indicate an approximate confidence interval. The p – value of each smoothed term is approximate, and indicates whether the change in deviance is significant compared to the model without the term, based on the estimated number of degrees of freedom (*df*). For each smoothed term that was found significant in the regression model, the estimated *df*, expressing the complexity in the shape, is included in addition to the approximate p – value in the tables and selected plots as illustrations. Due to sparse data, a spline surface of two covariates simultaneously was only moderately successful. Instead, linear interactions were fitted. Extreme values were found in some of the incidence rates. These outliers, from one to four, had unreasonable impact on the estimation and were removed from the analysis.

A number of different confounders (e.g. demographics) were chosen as covariates in the regression models, and model selection was based on the statistically significant ones.

## Results

### Factor analysis of CTQ

A three – factor solution was found sufficient and similar to the models with five factors. We named them Factor 1 = emotional abuse/neglect-revised, Factor 2 = sexual abuse-revised, and Factor 3 = physical abuse-revised. Factor loadings for the rotated solution are shown in Table 2.

**Table 2:** Factor loadings for rotated solution (three factors)

**CTQ item**^**a**^	**Factor 1**	**Factor 2**	**Factor 3**
There was not enough food in the house for everyone CTQ **001**	.370	.145	.300
I knew there was someone to take care of me and protect me ^b^**CTQ 002**	.736	.136	.154
People in my family said hurtful or insulting things to me **CTQ 003**	.505	.124	.325
My parents were to drunk or high to look after the family **CTQ 004**	.328	.245	.148
There was someone in my family who helped me feel that I was important or special ^b^**CTQ 005**	.530	.132	.088
I had to go in dirty cloths **CTQ 006**	.320	.013	.202
I felt loved ^b^**CTQ 007**	.807	.208	.112
I thought that my parents wished I had never been born **CTQ 008**	.539	.101	.278
Someone in my family hit so hard I had to go to the doctor or the hospital **CTQ 009**	.244	.292	.452
People in my family hit me so hard that it left me with bruises or marks **CTQ 011**	.257	.071	.819
I was punished with a belt, a board, a cord, or some other hard objects **CTQ 012**	.192	.102	.596
People in my family looked out for each other ^b^**CTQ 013**	.856	.003	.117
People in my family said hurtful or insulting things to me **CTQ 014**	.623	.047	.402
I believe I was physical abused **CTQ 015**	.309	.164	.802
I got hit or beaten so badly that it was noticed by someone like a neighbor, teacher, or doctor **CTQ 017**	.153	.147	.715
I felt someone in my family hated me **CTQ 018**	.572	.122	.375
People in my family felt close to each other ^b^**CTQ 019**	.692	.092	.062
Someone tried to touch me in a sexual way or tried to me make touch them **CTQ 020**	.177	.873	.062
Someone threatened to hurt me or tell lies about me unless I did something sexual with them **CTQ 021**	.170	.634	.132
Someone tried to make me do sexual things or watch sexual things **CTQ 023**	.112	.928	.083
Someone molested me **CTQ 024**	.084	.770	.261
I believe I was emotional abused **CTQ 025**	.664	.145	.439
Someone took me to a doctor if I needed ^b^**CTQ 026**	.445	.104	.182
I believe I was sexually abused **CTQ 027**	.071	.906	.103
My family was a source of strength and support ^b^**CTQ 028**	.852	.086	.225

^a^ Each item begins with the phrase “ when I was growing up”, and is rated on a 5 point Likert-type scale. Response options are “never true”, “rarely true”, “sometimes true”, “often true”, and “very often true”.

^b^ Reverse-coded item.

This is fewer factors than reported earlier [24], 1994, but they have a comparable amount of explained variance, 53.3%. By reducing the number of factors from five to three, we were able to reduce the complexity of the model.

### Childhood trauma and clinical characteristics

Associations between childhood trauma (CTQ total and factor scores) and different continuous outcome variables (age of onset and Global Assessment of Functioning [GAF-S and GAF-F] scores) are presented in Table 3, and Additional file 1: Figure S1.

**Table 3:** Regression (cox and linear) for continuous outcomes with significant influence of total CTQ score or factor scores, in a sample of bipolar patients

**Outcome**	**Covariate**	**Estimate (st.error)**	**p-value**
Age of onset	CTQ total	HR^#^ = 1.029 (0.007)	<0.001
Age of onset	Gender, male	1 (Ref)	
	Female	HR^#^ = 1.57 (0.47)	0.016
	Factor 1	HR^#^ = 1.33 (0.15)	<0.001
GAF-F	Intercept	53.56 (1.66)	<0.001
	CTQ total	−0.16 (0.076)	0.032
GAF-F	Intercept	50.82 (1.07)	<0.001
	Factor 1	−2.08 (1.03)	0.046
	Factor 3	−2.27 (1.08)	0.038
GAF-S	Intercept	56.75 (1.66)	<0.001
	CTQ total	−0.14 (0.075)	0.059

#: HR = Hazardrate Ratio. Factor 1 = emotional abuse/neglect-revised; Factor 3 = physical abuse-revised.

Higher CTQ total score, as well as increased emotional abuse/neglect-revised factor score, were associated with earlier age at onset. An increase of one point on the CTQ total score represents a significant increase in the hazard of onset by 2.9% (hazard ratio = 1.029, p < 0.001). This is interpreted as an increased risk of onset at a given time – point of 2.9% for an extra point of the CTQ total score. One unit increase in the emotional abuse/neglect-revised factor score represented an increased risk of 33% (hazard ratio = 1.33, p < 0.001).

GAF-F was significantly associated with CTQ total and factor scores emotional abuse/neglect-revised and physical abuse-revised. For an increase of one unit of the CTQ total score GAF-F decreased by 0.16 (p = 0.032). One unit increase of emotional abuse/neglect-revised or physical abuse-revised scores were associated with a decrease in Global Assessment of Functioning (GAF-F) by 2.08 (p = 0.046) and 2.27 (p = 0.038), respectively. Global Assessment of Symptoms (GAF-S) also showed a strong trend towards association with CTQ total score as shown by a decrease of 0.14 for one unit increase in the CTQ total score (p = 0.059).

The association between childhood trauma (CTQ total score and factor scores) and number of mood episodes, and self – harm episodes are shown in Tables 4, 5.

**Table 4:** Regression for incidence rates (with overdispersion and offset term) of various types of episodes with significant influence of total CTQ score, in a sample of bipolar patients

**Outcome**	**Covariate**	**Estimate (st.error)**	Estimated df^**^	**p-value**
Depressive episodes	Intercept	−1.43 (0.29)		<0.001
	Gender, male	0 (Ref)		
	female	0.36 (0.16)		0.027
	s(age)^*^		3.8	<0.001
	s(CTQ total)^*^		2.9	0.054
Hypo/manic episodes	Intercept	−1.61 (0.31)		<0.001
	Gender, male	0 (Ref)		
	female	0.43 (0.18)		0.017
	s(age)^*^		8.63	<0.001
	s(CTQ total)^*^		7.4	0.085
Psychotic episodes	Intercept	−2.13 (0.15)		<0.001
	s(age)^*^		1.33	<0.001
	s(CTQ total)^*^		8.73	0.038
Self – harm (N=123	Intercept	−7.15 (1.43)		<0.001
	Gender, male	0 (Ref)		<0.001
	female	2.82 (0.72)		<0.001
	s(age)^*^		3.8	<0.001
	s(CTQ total)^*^		6.19	0.011
Hospitalizations	age	−0.04 (0.004)		<0.001
	CTQ total	−0.019 (0.008)		0.01

*: s(.) = smoothed(.).

**: Estimated df corresponds to amount of smoothing (approximate p-value).

**Table 5:** Regression for incidence rates (with overdispersion and offset term) of various types of episodes with significant influence of factor scores, in a sample of bipolar patients

**Outcome**	**Covariate**	**Estimate (st. error)**	df^**^	**p-value**
Depressive episodes	Intercept	−1.5 (0.3)		<0.001
	Gender, male	0 (Ref)		
	female	0.4 (0.17)		0.02
	s(age)^*^		4.7	<0.001
	s(Factor 3)^*^		6.6	0.04
Hypo/manic episodes	Intercept	−1 (0.09)		<0.001
	s(age)^*^		8.7	<0.001
	s(Factor 1)^*^		8.3	<0.001
	age*Factor 1	−0.025 (0.008)		0.001
Psychotic episodes	Intercept	−2.2 (0.16)		<0.001
	s(age)^*^		1.65	0.001
	s(Factor 2)^*^		2.8	0.009
	age*Factor 2	−0.032 (0.016)		0.042
Self – harm (N=123)	Intercept	−5.56 (1.56)		<0.001
	Gender, male	0 (Ref)		
	female	3.08 (0.6)		<0.001
	age	−0.064 (0.02)		0.002
	s(Factor 3)^*^		7.8	<0.001
	age*Factor 3	0.19 (0.03)		<0.001
Hospitalizations	age	−0.048 (0.003)		<0.001
	Factor 1	−0.33 (0.11)		0.003

*: s(.) = smoothed (.).

**: Estimated df corresponds to amount of smoothing (approximate p-value).

Factor 1 = emotional abuse/neglect-revised; Factor 2 = sexual abuse-revised; Factor 3 = physical abuse-revised.

Significant non – linear associations were found between CTQ total score and incidence of psychotic episodes (df = 8.73, p = 0.038) and self – harm episodes (df = 6.19, p = 0.011). Nearly significant non – linear associations were found between CTQ total score and incidence of depressive episodes (df = 2.9, p =0.054) and (hypo) manic episodes (df = 7.4, p =0.085; see Additional file 1: Figure S2). Even though these associations improved the model fit, none of them were clearly uni – directional. A linear association between CTQ total and number of hospitalizations was also observed (linear slope coefficient = - 0.019, p = 0.01).

The decomposition of the CTQ score by factor analysis revealed that different factors were associated with different incidence rates. Emotional abuse/neglect-revised factor score was significantly non – linearly associated with incidence of (hypo) manic episodes (df = 8.3, p < 0.001), and linearly associated with number of hospitalizations (slope coefficient = -0.33, p=0.003). The sexual abuse-revised factor score was significantly non – linearly associated with incidence of psychotic episodes (df = 2.8, p = 0.009). The physical abuse-revised factor score was significantly non – linearly associated with incidence of depressive episodes (df = 6.6, p = 0.04), as well as incidences of self – harm episodes (df = 7.8, p < 0.001).

## Discussion and conclusion

Our data suggests that childhood trauma is significantly associated with a more severe form of bipolar illness. Our data also demonstrate that specific subtypes of trauma (physical, sexual or emotional abuse/neglect) are associated with different clinical characteristics of the disorder. Patients who reported increased incidences of childhood trauma presented earlier age of onset, compared to patients reporting less severe, or no childhood trauma experience. For psychotic episodes, as well as self-harm, associations were characterized by fluctuations and large variance, without being clearly uni – directional. Secondly, we also found that childhood trauma total scores were significantly related to lower current functioning (lower Global Assessment of Functioning [GAF-F] scores), compared to patients reporting less severe, or no childhood trauma. This is to our knowledge the first time childhood trauma has been linked to lower Global Assessment of Functioning (GAF-F) and Global Assessment of Symptoms (GAF-S) scores in patients with bipolar disorder. Thirdly, three dimensions were found to be a sufficient description of the total CTQ score: the emotional abuse/neglect items, sexual abuse only, and physical abuse only factor scores. Each of these factors was found to be significantly associated with the illness. The emotional abuse/neglect factor score was the only factor associated with earlier onset. Based on the association between CTQ total score and early onset, we may hypothesize that this in fact is due to the underlying effect of emotional abuse/neglect factor on age of onset. The emotional abuse/neglect factor, together with the physical abuse-revised factor, were also significantly and linearly associated with reduced functioning Global Assessment of Functioning (GAF – F). The factor scores were all separately associated with one or more of the incidence rates. Again, these significant non – linear associations were characterized by fluctuations and large variance, both for depressive and (hypo) manic episodes, making them non – conclusive with respect to direction. We also observed a negative association between both CTQ total score and the emotional abuse/neglect factor score and number of hospitalizations: The higher the trauma score the lower the number of hospitalizations. The emotional abuse/neglect factor score includes emotional neglect, of which a high level represents little commitment from near relatives to monitor and help the patient. It might be that experiencing high levels of neglect as a child also influence the quality of interpersonal relationships in adulthood. Thus, these patients may lack assistance from close relatives or friends when needed, which in combination with lower social functioning, may prevent them from getting optimal hospital care, when needed (it is worth mentioning that all patients were recruited from a well functioning welfare community with equal access to support and help).

The association between childhood trauma total score and early onset was also found in previous studies (e.g. [6]). However, in contrast to previously published papers (focusing on physical abuse and sexual abuse), we found that childhood emotional abuse/neglect was related to a reduced number of hospitalisation, instead of an increased incidence rates as reported in previous literature. Furthermore, our data show that not only childhood physical abuse and sexual abuse, but also emotional abuse and neglect are predictors of unfavourable bipolar disorder outcomes. Our study is also, to our knowledge, the first to show that childhood trauma is linked to reduced functioning, shown by lower GAF scores in patients with bipolar disorder.

There are several possible mechanisms behind our findings. Firstly, childhood trauma has been associated with persistent sensitization and increased activation of the Hypothalamic Pituitary Adrenal (HPA) axis [26]. Secondly, severe stress, like the one seen in childhood trauma, affects both structure and function of brain areas important for cognition and emotional processes, such as hippocampus, amygdala, and the prefrontal lobe [27,28]. This is supported by theories of the central nervous system (CNS) being more “plastic” in the earlier years of childhood, which consequently may lead to a more profound influence of adverse events during this period. This is supported by studies from non-human primates: In rodents it has been demonstrated that neurobiological changes, leading to increased vulnerability to stress in adulthood occur when young animals are deprived of maternal care during their post natal period [11]. Similar findings have also been observed in humans [29]. These neurobiological changes may point to an increased risk of bipolar disorder, and a more severe cause of the illness, since childhood trauma possibly may lead to disturbances in affective regulations [15,30]. However, as already pointed out by Etain et al. 2008, no study has so far definitely demonstrated causality between childhood trauma and bipolar disorder [15].

Strengths of this study are that we studied a representative sample of patients with bipolar disorder (n=141) recruited from both psychiatric in- and out-patient clinics in one catchment area. Besides, we used *one* standardized operationalized instrument (CTQ) to measure total scores, as well as different subtypes of trauma, which were reduced to three by factor analysis. A further novel contribution of the current study is its use of factor analysis on the CTQ reducing the factors from five to three with the same explained variance of 53.3% as in the original factor analysis [24]. By reducing the number of factors, we were able to reduce the complexity of the model.

Several limitations to our study should be noted: The childhood abuse data rely on retrospective self-report of abuse history. Although the CTQ is a validated instrument for collecting such data, there are inherent weaknesses in its retrospective reporting design. However, the retrospective collection of childhood trauma in patients with severe mental disorders has been found a valid and reliable source to obtain information in previous studies [16,31]. Furthermore, we did not take into consideration euthymic states or current mood symptoms when the patients were assessed. This may have led to recall biases since current mood symptoms may lead to under or over reporting of traumatic events [5]. Having said this, all patients from the Norwegian sample underwent a lengthily in-depth interview, which was not suited for patients in severe current pathological affective states; therefore when necessary, patients were re-contacted to continue the interview at a later time when they were able to consent to the study, as well as able to fill in the CTQ questionnaire. Also in retrospective studies where current mood is corrected for, studies conclude that patients with bipolar disorder have higher prevalence of childhood trauma [5,15], indicating that our findings can not only be explained by current mood. Moreover, supporting this are recent neuroimaging studies also showing that the key factor to reports of past trauma exposure are not based on current mood symptoms, but possibly to long-term changes of the Hypo-Thalamic-Pituitary-Adrenal (HPA) axis. For instance, Teicher et al. 2012 recently reported in a large group of young adults that there was a strong association between severity of self-reported exposure to abuse (using CTQ and ACE scores) and hippocampal subfields containing dentate gyrus and CA3 [32], which are the most stress-sensitive components of the hippocampus. This association was unrelated to whether or not they had a history of depression, PTSD, or severity of current mood symptoms. This may indicate, that our findings may be valid, independent of the patient’s current mood during evaluation.

Another limitation to our study was that we did not have data on childhood trauma for the healthy control group at time present. However, previous studies indicate that patients with severe mental illnesses, such as bipolar disorder report significantly more experiences of childhood trauma than the general population, possibly making them more vulnerable for the negative effects of trauma [5,15]. Our study also lacks specific information regarding timing of the traumatic event(s) in childhood. We know for example from imaging studies that childhood trauma exposure at specific time points during childhood have different effects on different parts of the brain [33,34]. The study by Andersen et al. 2008 demonstrate that a key sensitive period for hippocampus was between 3-5 years, while prefrontal cortex have a sensitive period between 14-16 years. This is supported by translational studies showing that synaptic density in hippocampus, but not prefrontal cortex is affected by maternal separation stress prior to weaning [33,35], while synaptic density in prefrontal cortex, but not hippocampus is affected by exposure to social stress during preadolescence [33]. Moreover, reports of exposure to sexual abuse prior to age 12, but not after, is associated with reduced gray matter volume in visual cortex [36], which again supports the need of more robust information on the time point of the traumatic event(s). This is concordant with the observation that visual cortex is highly plastic up until puberty in primates [37]. Future studies on the long-term effects of childhood trauma should therefore, based on the above, also include information regarding time-points of the childhood trauma event(s). Lastly, our sample was relatively small which affects the generalizability of the results.

Our results suggest that childhood trauma is associated with earlier onset of bipolar disorder and several important clinical characteristics of the illness in adulthood. Further, childhood abuse (physical and sexual), as well as emotional abuse/neglect are significantly associated with accelerating staging process of bipolar disorder. Lastly, using specific trauma subtypes (physical abuse, sexual abuse or emotional abuse/neglect), associations become both more precise and diverse.

## Competing interests

The authors declare that they have no competing interests.

## Authors’ contributions

SLa and SLo planned and designed the study in this paper and IA, IM, OAA planned and designed the main research project, that this paper is a part of. SLa, MA, EM, NES, EAB, TVL and JIR have taken part in the interviews and data collection and OK have carried out all statistical analyses. All authors have contributed in developing the manuscript and have read and commented on several versions. All have also read the final version and are responsible for the content. All authors read and approved the final manuscript.

## Pre-publication history

The pre-publication history for this paper can be accessed here:

http://www.biomedcentral.com/1471-244X/13/97/prepub

## Supplementary Material

Additional file 1: Figure S1 Linear associations between total CTQ score and clinical characteristics of bipolar disorder, log-hazard rate. **Figure S2.** Non-linear associations between CTQ total score and symptoms (with approximate confidence intervals), with smoothing by generalized additive models (GAM). Click here for file
