# Supplementary material for: Patterns of childhood adverse events are associated with clinical characteristics of bipolar disorder
Source: BMC Psychiatry. 2013 Mar 22;13:97. doi: 10.1186/1471-244X-13-97 (PMC3637635; doi:10.1186/1471-244X-13-97)

**Online Supplementary Material**

**Figure 1:** Linear associations between total CTQ score and clinical characteristics of bipolar disorder, log-hazard rate


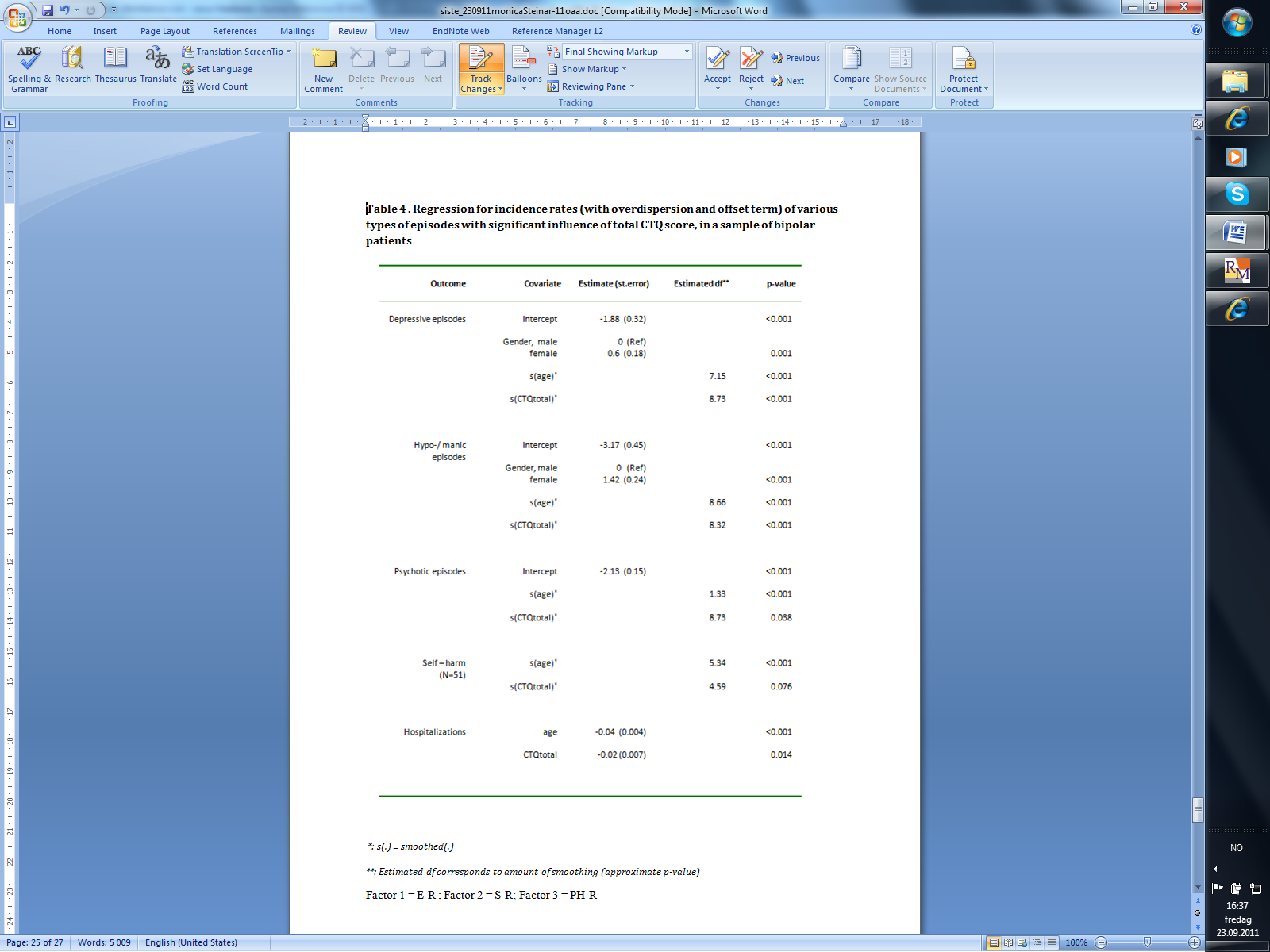


CTQ total and Age of onset

CTQ total and GAF-F

**Figure 2:** Non-linear associations between CTQ total score and symptoms (with approximate confidence intervals), with smoothing by generalized additive models (GAM**)**
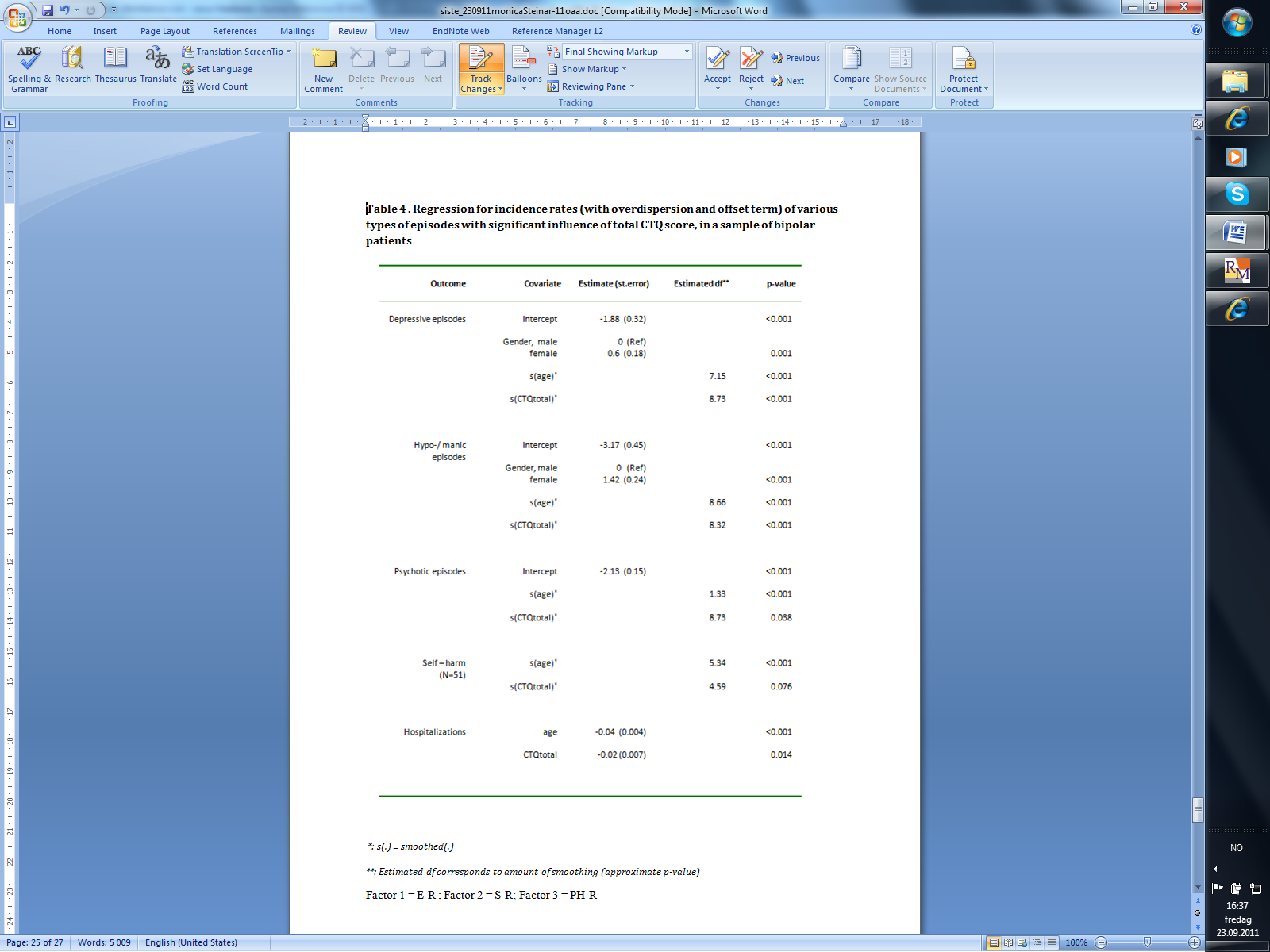

Supplement: Additional file 1: Figure S1 — Linear associations between total CTQ score and clinical characteristics of bipolar disorder, log-hazard rate. Figure S2. Non-linear associations between CTQ total score and symptoms (with approximate confidence intervals), with smoothing by generalized additive models (GAM). [file 1471-244X-13-97-S1.doc]
